# Understanding Nanoparticle Toxicity to Direct a Safe-by-Design Approach in Cancer Nanomedicine

**DOI:** 10.3390/nano10112186

**Published:** 2020-11-02

**Authors:** Jossana A. Damasco, Saisree Ravi, Joy D. Perez, Daniel E. Hagaman, Marites P. Melancon

**Affiliations:** 1 Department of Interventional Radiology, The University of Texas MD Anderson Cancer Center, Houston, TX 77030, USA; jdamasco@mdanderson.org (J.A.D.); jdperez@mdanderson.org (J.D.P.); dhagaman76@gmail.com (D.E.H.); 2 School of Medicine, University of Texas Rio Grande Valley, Edinburg, TX 78539, USA; saisree.ravi01@utrgv.edu; 3 UT Health Graduate School of Biomedical Sciences, The University of Texas MD Anderson Cancer Center, Houston, TX 77030, USA

**Keywords:** nanotoxicity, cancer nanomedicine, inorganic nanoparticles

## Abstract

Nanomedicine is a rapidly growing field that uses nanomaterials for the diagnosis, treatment and prevention of various diseases, including cancer. Various biocompatible nanoplatforms with diversified capabilities for tumor targeting, imaging, and therapy have materialized to yield individualized therapy. However, due to their unique properties brought about by their small size, safety concerns have emerged as their physicochemical properties can lead to altered pharmacokinetics, with the potential to cross biological barriers. In addition, the intrinsic toxicity of some of the inorganic materials (i.e., heavy metals) and their ability to accumulate and persist in the human body has been a challenge to their translation. Successful clinical translation of these nanoparticles is heavily dependent on their stability, circulation time, access and bioavailability to disease sites, and their safety profile. This review covers preclinical and clinical inorganic-nanoparticle based nanomaterial utilized for cancer imaging and therapeutics. A special emphasis is put on the rational design to develop non-toxic/safe inorganic nanoparticle constructs to increase their viability as translatable nanomedicine for cancer therapies.

## 1. Introduction

The World Health Organization estimates 9.6 million people died from cancer in 2018. That is one in six deaths, which makes it the second leading cause of death worldwide [1]. American Cancer Society estimates the United States will have approximately 1,806,590 new cancer cases and 606,520 cancer deaths in 2020 [2]. A major challenge in effectively treating cancer is its intratumor heterogeneity brought about by mutations as the disease progresses. These considerable variations among tumors can lead to different responses to a given treatment and may lead to ineffective killing of particular subclonal populations [3,4]. Conventional cancer treatments rely on surgery, chemotherapy and radiotherapy. Most often, combinations of these therapies are needed to completely eradicate the disease. However, healthy tissues are also affected by these treatments resulting to adverse side effects. [5]. As such, design of more effective cancer therapies will require careful planning and integration of diagnosis and therapy to tailor treatments to individual needs. Nanomedicine, which is the application of nanotechnology to the diagnosis, treatment, and prevention of disease, offers to address shortcomings of conventional treatment in cancer through various biocompatible nanoplatforms with diversified capabilities for tumor targeting, imaging, and therapy. Emerging nanotechnologies are geared towards imparting imaging functions in addition to their therapeutic capabilities in order to yield individualized therapy that can be monitored non-invasively and in real time [6]. The increasing need for novel therapeutics has led to dramatic growth in the development of therapeutics and imaging agents based on inorganic-based nanoparticles, such as gold, silica, iron oxide, copper, zinc, bismuth, gadolinium, etc. Nanosized materials exhibit unique properties compared to their bulk counterparts, including high surface-to-volume ratio, high surface energy, unique mechanical, thermal, electrical, magnetic, and optical behaviors, can be tailored to suit a specific application and make them a truly multifunctional platform for imaging and therapy [7]. In addition, inorganic nanoparticles are extremely robust and highly resistant to enzymatic degradation [8,9]. They can be engineered to a controlled size to improve delivery, distribution, and clearance [10].

The European Medicines Agency (EMA) defines nanomedicine as purposely designed systems for clinical applications, with at least one component at the nanoscale size, with definable specific properties and characteristics related to the nanotechnology application, associated with the expected clinical advantages of the nanoengineering, and needs to meet definition as a medicinal product according to European legislation [11,12]. Although the Food and Drug Administration (FDA) does not have its own definition, it has adopted the commonly used terms of having at least one dimension in the ~1–100 nm size range, and advises that evaluations should consider any unique properties and behaviors that the application of nanotechnology may impart [13]. Despite the many advances from the bench, very few clinical applications of inorganic nanoparticle-based nanomedicine in cancer exist [14,15]. As the unique properties brought about by the small size of the nanoparticles offer great opportunities for medical purposes, at the same time, safety concerns have emerged as their physicochemical properties can lead to altered pharmacokinetics, with the potential to cross biological barriers. In addition, the intrinsic toxicity of some of the inorganic materials (i.e., heavy metals) and their ability to accumulate and persist in the human body has been a challenge to their translation. Successful clinical translation of these nanoparticles is heavily dependent on their stability, circulation time, access and bioavailability to disease sites, and their safety profile [14]. Thus, rational design to tailor for specific applications, to optimize their pharmacokinetic parameters, and to minimize off-target toxicity, is critical in moving these constructs into the clinic. This review covers preclinical and clinical inorganic-nanoparticle based nanomedicine utilized for cancer imaging and therapeutics. A special emphasis is put on the rational design to develop non-toxic/safe inorganic nanoparticle constructs to increase their viability as translatable nanomedicine for cancer therapies.

## 2. Inorganic Nanoparticles in Cancer Nanomedicine

**Figure 1:**
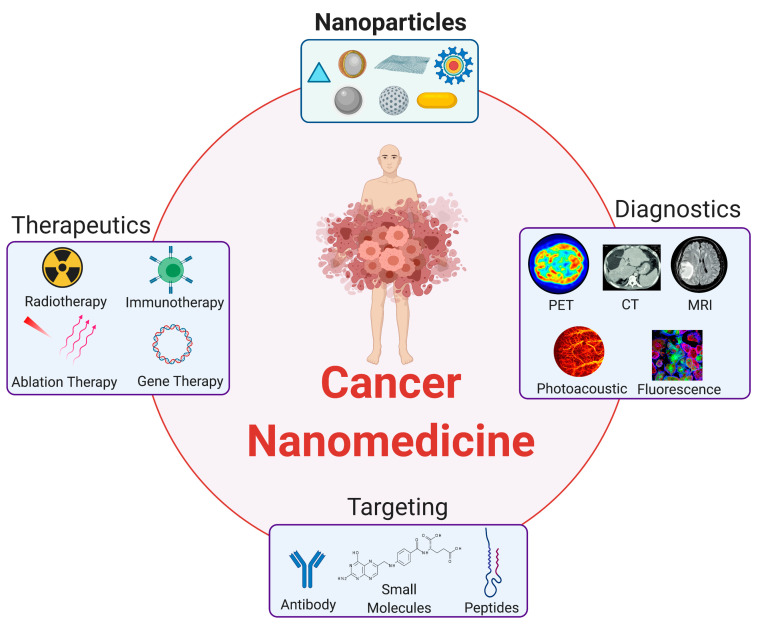
Nanoparticles in imaging and therapies. Created using BioRender.com.

The compatibility between nanoparticle size and the biological systems, coupled with the ability to tailor their physicochemical properties with thorough characterizations facilitated the rapid rise of nanoparticles as unique tools for many biomedical applications (Figure 1). These nanoparticles engineered with multifunctionalities showing great promise to a more personalized approach to disease management and therapies could considerably improve the diagnostics and therapeutics of various cancers [16].

Imaging is largely considered as one of the most relied-upon diagnostic tools in healthcare. Through its unique nanoparticle surface or core features, a nanoplatform can provide contrast enhancement capabilities in various imaging methods for purposes of early detection, screening, diagnosis, and image-guided cancer treatment. These imaging techniques include optical, plain radiography, magnetic resonance imaging (MRI), and computed tomography (CT). The possibility to introduce multiple modalities in the same nanoparticle with minimal interference makes nanoplatforms both attractive and advantageous. Such nanoplatforms can effectively function as multimodal contrast agents for imaging to give complementary information for a precise diagnosis.

In addition to multimodality, nanoparticles are also being developed to complement conventional therapeutic avenues, such as surgery, chemotherapy, ablation, and radiation therapy [7]. As the technology progresses, multimodality and multifunctional therapies have emerged. A single agent capable providing contrast enhancement to different imaging modalities can provide a more accurate and detailed information on the physiological and anatomical characteristics of the disease pathology. Adding imaging with therapeutic delivery achieves a safer and more effective approach since sufficient accumulation in target tissues can be ensured and the effects both on the target and the surrounding healthy tissues can be monitored.

### 2.1. Imaging Modalities

#### 2.1.1. Positron Emission Tomography

Positron emission tomography (PET) is a 3D-imaging method that makes use of low energy γ rays via a positron-emitting radionuclide in the imaging contrast agents. Nanoparticles are particularly useful in the development of new contrast agents. In particular, PET imaging can be performed at a much lower dose by attaching an increased number of radionuclides on the surface of the nanoparticle. Incorporation of the radionuclide in a non-radioactive nanomatrix results in its radioactive functionality. The dosage and type of radionuclide is determined such that emitted species is of sufficiently low energy as to prevent concurrent radiotoxicity, while providing reliable radioimaging signals. The most commonly used method of nanoparticle radiolabeling includes surface chelation of the radionuclide and using chelator-free post-synthetic labeling via ion-exchange [17]. A key advantage to radiolabeling of nanoparticles is the possibility of accurate earlier tumor detection while providing mechanism of cancer proliferation at the molecular scale. For example, poly(aspartic acid)-iron oxide (Fe_3_O_4_) nanoparticles (IONP, 5-nm) can be functionalized with both ^64^Cu for PET, and cyclic arginine-glycine-aspartic (RGD) peptides for targeting integrin α_v_β_3_. This is achieved by reacting the amino groups on the surface of the nanoparticles with activated *N*-hydroxysuccinimide (NHS) macrocyclic 1,4,7,10-tetraazacyclododecane-*N,N′,N″,N‴*-tetraacetic acid (DOTA) chelator for ^64^Cu labeling, and with NHS−poly(ethylene glycol) (PEG)−maleimide for RGD peptide functionalization. The resulting bifunctional IONP imaging probe allows the simultaneous PET and MRI scans of tumor integrin α_v_β_3_ expression [18]. Click chemistry has also been used to attach large number of ^18^F atoms in newly developed contrast agents resulting to drastic lowering of the detection threshold [19]. However, quick conjugation of ^18^F into the probe with high yield is essential due to its short half-life (t_1/2_ = 110 min). To be used for PET imaging, ^18^F can be ion-exchanged with the fluoride in NaYF_4_ upconversion nanoparticles through simple sonication. This facile method yielded more than 90% even after 2h of incubation [20]. Organically modified silica (ORMOSIL) radiolabeled with ^124^I (t_1/2_= 4.2 days) have been synthesized to allow whole body PET imaging. The ^124^I radioisotope was introduced on to the ORMOSIL surface by acylation with the Bolton–Hunter reagent [21].

#### 2.1.2. Magnetic Resonance Imaging

Magnetic resonance imaging (MRI) is a versatile imaging technique that offers broad applications and has fast become a routine tool for diagnosis both in the clinical and biomedical settings [22,23,24]. Among the advantages of MRI over other clinically non-invasive imaging modalities include its exceptional capability in discriminating between normal and pathological tissues, lack of beam hardening artifacts, use of non-ionizing radiation, and ability to image any plane with equivalent resolution without moving the patient [25,26]. Commonly, T1-weighted MRI utilizes T1 contrast agents for positive contrast enhancements, while T2-weighted MRI employs T2 contrast agents for negative contrast enhancements [27,28].

Engineering the surface of the nanoparticles with large numbers of paramagnetic centers can yield superior T1 contrast agents in comparison to clinical Gd-chelates. Nanoparticle T1 contrast agents can have higher T1 relaxivity [29,30,31,32,33,34,35,36] and longer blood circulation time [29,34,35,37]. In addition, the Gd^3+^ ions are embedded in the crystal matrix of the nanoparticle; hence, significant leaching of Gd^3+^ can be prevented, minimizing the risk of toxicity [29,35,36]. Another big advantage of the nanoparticles is its highly reactive surface that can be coated with functional biocompatible materials to allow conjugation for targeted delivery to the region of interest without compromising Gd^3+^ binding site [32,36]. To achieve ultrahigh T1 relaxivity requires precise control of the nanoparticle surface structure. A T1 relaxivity as high as ~80 mM^−1^·s^−1^ per Gd^3+^ was achieved at 1.41 T with ~3 nm NaGdF_4_ coated with high amount of PEGylated phospholipid (i.e., DSPE-PEG). Increasing the ratio of phospholipid to the nanoparticle will result in compact micellization (hydrodynamic diameter <5 nm), drawing the water protons closer to the Gd^3+^ surface [38]. To circumvent the potential toxicity risks of Gd^3+^, applicability of MnO_2_ nanoparticles as T1 contrast agents are also being explored. MnO_2_ gradually decomposes in an acidic environment (i.e., pH 6.8 in tumor microenvironment) releasing free Mn^2+^ [39]. Reduction of MnO_2_ to Mn^2+^ result in increased T1 relaxivity, which has been exploited in MnO_2_-based pH sensitive MRI probes and tumor microenvironment modulation [40,41,42,43].

IONPs, with their inherent superparamagnetic properties serve as excellent T2 contrast agents in MRI. The high magnetic moment of IONP reduces the transverse relaxation time of protons through an increased loss in phase coherence, reducing the MRI signal leading to a negative contrast [44,45]. Control of size and metal doping can produce IONP with large saturated magnetization (Ms values of 175 emu g^−1^ [46] and 186 emu g^−1^ [47]), while morphology control can tune the effective magnetic core radius, leading to a significant increase of T2 relaxivity. For example, octapod-shaped IONP achieved T2 relaxivity as high as 679.3 ± 30 mM^−1^ s^−1^, which is 5-fold higher than spherical IONP of similar volume (125.86 mM^−1^ s^−1^) [48]. An optical probe can also be incorporated with IONPs for dual MRI/optical imaging such as quantum dots (QDs) [49,50,51,52] or IONPs integrated with gold-based nanoparticle such as gold nanoshell (IONP@AuNS) to add therapeutic functionality [53,54]. Various formulations of IONPs have already been used in clinical MRI [55].

#### 2.1.3. Computed Tomography

CT is a non-invasive diagnostic tool primarily used for 3D visual reconstruction and segmentation of tissues of interests [56,57,58]. Array detector technology allowed for fast CT imaging (within several minutes) of whole body or organs with isotropic resolution at the sub-millimeter level [56]. This technique is fast, accurate, non-invasive, and painless, which can be performed on every region of the body. For these reasons, CT has become a valuable tool for diagnosis, treatment planning, and intervention and has become one of the most prevalent diagnostic devices in terms of frequency-of-use and hospital availability [59,60,61].

To improve visualization and enhance differentiation among adjacent tissues, CT contrast agents are generally given to increase X-ray attenuation [62]. The currently approved clinical intravenous contrast agents for CT imaging are iodinated small molecules [63]. However, their short blood half-life requires high dose concentrations that may cause contrast-induced nephropathy in patients with compromised kidneys [64]. Therefore, there is a need to find alternative contrast agents that require lower concentration dose and have minimal toxicity, while still providing sufficient signal enhancement. Nanoparticles containing elements with high atomic number (high-Z) are attractive alternatives to iodinated small molecules as they have higher X-ray attenuation and longer half-lives, and therefore can achieve high-quality CT imaging at lower contrast agent dose [65,66]. Additionally, the nanoparticle surface can be easily functionalized to enable targeted delivery to specific tissues [67]. Some of the promising high-Z nanoparticles being explored contains bismuth (Bi, Z = 83), tantalum (Ta, Z = 73), tungsten (W, Z = 74), gadolinium (Gd, Z = 64), and ytterbium (Yb, Z = 70) [68,69,70,71,72,73]. Nevertheless, gold-based nanoparticles (Au, Z = 79) have received the most attention due to their known excellent biocompatibility, ease of synthesis, and high X-ray attenuation compatible with clinical CT [74,75,76]. The first reported spherical gold nanoparticles (AuNP) administered in vivo for CT demonstrated 3-fold higher X-ray attenuation than the iodine-based contrast agent Omnipaque^TM^ [77]. Furthermore, these nanoparticles have ~1.9 nm diameters, thus can be cleared through renal filtration. Targeted CT imaging using gold nanorods (AuNR), coated with PEG and functionalized with anti-EGFR (epidermal growth factor receptors) on the surface to target head and neck cancer, exhibited more than 5 times higher X-ray attenuation in cancer cells than non-targeted AuNR [78]. Another example is AuNP with tunable diameter from 1.9–4.7 nm embedded within PEG-modified polyethylenimine (PEI) network for blood pool and tumor imaging have shown blood half-life of 11.2 h in rats [79]. Commercial formulation of 1.9 nm AuNP is available under the trade name AuroVist (Nanoprobes), which can be applied in micro-CT, clinical CT, planar CT, and mammography.

Nanoparticles containing two or more CT-contrast elements to provide high X-ray attenuation at different X-ray operating voltages have also been synthesized. BaYbF_5_ nanoparticles (Ba, Z = 56 and Yb) with SiO_2_ shell and PEGylated surface (BaYbF_5_@SiO_2_@PEG) demonstrated higher X-ray attenuation than the clinical tri-iodinated iobitridol and the single CT-contrast element NaYbF_4_, at both 80 and 140 kVp Xray voltages [80].

#### 2.1.4. Optical Imaging

Interests in developing optical imaging probes, in particular for tumor margin delineation to guide surgical resections, arise from certain limitations of current clinical imaging methods such as narrow imaging time windows, suboptimal specificity, and sensitivity. Near infrared (NIR) imaging is a technique that offers high spatial resolution while maintaining a less expensive and non-invasive solution. In the last years, the use of NIR fluorescence has gained a lot of traction for bioimaging purposes for its superior penetration in biological tissues. Furthermore, improved signal to noise ratio is achieved via reduced light scattering and minimized autofluorescence, having both the excitation and emission wavelengths within the optical transparency window. This technique not only allows for diagnosis at the cellular and single molecular levels but also provides imaging guidance for intraoperative surgical excision of tumors [81].

For in vivo tumor targeting studies, heavy metal-free QDs are particularly appealing due to their low toxicity risk common in heavy metals. Photoluminescent Si-based nanoparticles (SiNP) have been coated with PEG and administered in mice for sentinel lymph node (SNL) mapping, and multicolor NIR imaging [82]. Ultrasmall fluorescent SiNP (~4 nm) in *Caenorhabditis elegans* had also demonstrated superior photostability than mCherry and outperformed CdTe QD over a 120 min continuous high-power laser photobleaching study [83]. Their remarkable photostability make them suitable for long term cancer cell tracking, showing stable fluorescence in tumor-bearing mice over 20 days [84]. Another example of silicon-containing nanoparticle used for optical imaging was the luminescent porous silicon nanoparticles, which are able to carry a drug payload and can be monitored optically in vivo for both their accumulation and degradation into renal clearable components [85].

Currently, silica nanoparticles with ultrasmall size of 6–7 nm—better known as Cornell dots or C dots, have been undergoing a FDA Investigational New Drug (IND) human clinical trial for real time mapping of nodal metastases [86]. These C dots are silica-organic hybrid consist of cyanine dye, Cy5, encapsulated in a silica core-shell, and further coated with PEG that can be functionalized with target molecules (i.e., cyclic arginine-glycine-aspartic acid (RGD), anti-HER2 scFv fragments), or chelators for radiolabeling (i.e.,^124^I, ^89^Zr). These fluorescent core-shell nanoparticles serve as cancer probes for surgeons by binding to overexpressed receptors on tumor surfaces and would fluoresce when exposed to near-infrared (NIR) light. These compact targeted optical-PET probes have great tumor selectivity and sensitivity, reduced off-target accumulation in the reticuloendothelial system (RES) or kidney over a 24 h period and are renal clearable without excessive kidney irradiation [86,87].

#### 2.1.5. Photoacoustic Imaging

Photoacoustic (PA) imaging is a hybrid technique that relies on the generation of a sound wave by a material after light absorption due to thermoelastic expansion. The choice of imaging contrast for PA modality is determined by the difference in absorption between the target and its surrounding area while the spatial resolution is scaled with the ultrasonic frequency. PA beneficially combines the higher contrast of optical imaging and the longer penetration depth of ultrasound. [88]. Nevertheless, the penetration depth of excitation photons restricts imaging depth.

Several PA active nanoparticles have been developed for preclinical application. For example, spherical hollow AuNS (HAuNS) have been used to monitor treatment effects using PA imaging in nude mice bearing 4T1 tumors [89], while Au nanocages (AuNC) have been used for SNL mapping as deep as 33 mm below the skin surface on a Sprague-Dawley rats [90]. Semiconductor copper sulfide (CuS) nanoparticles with broad absorption at 1064 nm were able to enhance rat lymph nodes 12 mm below the skin surface [91]. A phantom study of these CuS nanoparticles in agarose gel showed it can be readily imaged when embedded in chicken breast muscle at ~5 cm deep. Another plasmonic semiconductor, PEGylated phospholipid coated copper selenide nanoparticles (Cu_2−*x*_Se NP) were evaluated for SNL mapping, achieving an imaging depth about 3.5 mm beneath the skin surface and with 4.8 times higher PA amplitude than the background [92]. Coupling these Cu_2-*x*_Se NP with AuNP to form heterodimer Au-Cu_2−*x*_Se NP resulted in a much-improved imaging depth up to 17 mm [93]. Molecular-specific detection of micrometastasis with enhanced specificity and sensitivity was developed utilizing molecularly activated plasmonic nanosensors (MAPS) specific for spectroscopic PA (sPA). MAPS that consist of EGFR-targeted AuNP produced a dramatic change in the spectroscopic signal of sPA imaging when they interacted with epidermoid carcinoma cells (A431) [94,95].

### 2.2. Therapeutic Modalities

#### 2.2.1. Radiation Therapy

Radiation therapy (RT) is a type of cancer treatment that utilizes ionizing radiation, most often X-rays, to control or kill cancer cells by damaging DNA [96]. RT is one of the most cost-effective treatments for cancer patients and remains an integral part in clinical oncology [97]. However, not all patients respond to RT and cancer recurrence is still a significant clinical problem [98]. To improve efficacy of RT, it is often administered in combination with chemotherapy (chemoradiation therapy) but this can increase systemic toxicity and even mortality [99]. One of the promising applications of inorganic nanoparticles is their ability to enhance radiotherapeutic efficacy. In this regard, there is growing interest in the use of high-Z nanoparticles as radiosensitizers. High-Z nanoparticles are known to enhance the photoelectric and Compton effects upon interaction with X-rays, leading to increased emitted secondary electrons. This dose radio-enhancement effect improves therapeutic efficacy in two ways: (1) radiation damage is enhanced without increasing the X-ray radiation dose, and (2) amplified DNA damage is localized to the tumor sparing the surrounding healthy tissues [97,100,101].

Most studies have focused on AuNPs and significant evidences have been reported to demonstrate their ability to increase the therapeutic ratio of radiotherapy [102,103,104,105]. Recently, it was shown that systemic administration of RGD conjugated to PEGylated AuNP (RGD:AuNP) in combination with image-guided RT lead to specific targeting of tumor blood vasculature. The site-specific damage of tumor endothelium can improve RT outcome with minimized off-target toxicities [106]. In addition, the alteration of the tumor blood vessels changes the vascular permeability of the tumor leading to improved drug delivery [107]. Another promising candidate for radiosensitizations are the Gd-based nanoparticles. Mesoporous silica nanoparticles (MSNs) loaded with Gd (Gd-MSNs) have shown great potential in inhibiting tumor when irradiated with a precisely tuned monochromatic X-ray [108]. This study has demonstrated two important factors regarding effective nanoparticle radiosensitization: (1) the energy compatibility between the binding energy of the nanoparticle and the irradiation source to maximize the dose radio-enhancement, and (2) the proximity of the nanoparticle to the nucleus of the target cell due to the low energy and consequent short-range characteristics of the generated Auger electrons. In this study, Gd-MSNs incubated in the human ovarian cancer (OVCAR8) accumulated in the lysosomes close to the nucleus have shown near complete destruction of tumor spheroids upon exposure to monochromatic 50.25 KeV X-rays. This strategy could enable highly targeted radiation therapy. In the clinic, first-in-class radioenhancer hafnium oxide (HfO_2_, Z = 72) developed by NanoBiotix (Paris, France), has already shown success as efficient radiation enhancers on patients requiring preoperative radiotherapy [109]. These 50 nm HfO_2_ nanospheres can substantially enhance radiation therapy efficacy when intratumorally injected [110]. Other metal nanoparticles that have shown great promise in augmenting radiotherapy include Bi [111,112], platinum (Pt, Z = 78) [112,113,114], and IONP [115,116] having both radiosensitizing and hyperthermic [117,118,119] properties.

#### 2.2.2. Ablation Therapy

##### Photothermal Therapy

Photothermal therapy (PTT) is a light-based therapy designed to eradicate tumors via conversion of light energy to heat through optical absorption [120]. Minimally invasive targeting of difficult-to-treat tumors can be achieved via selective photothermal absorbers. Nonetheless, because of its limited penetration depth, the use of this treatment modality is restricted only to cancers that are close to the skin, to internal linings that are accessible by endoscopy, or to organ surfaces exposed during surgery [6]. The photothermal absorbers must have an optimal absorbance within the first (650–850 nm) or the second (950–1350 nm) biological window, where light can penetrate deeply into the tumors due to the minimal attenuation from healthy tissues [121]. Due to their exemplary optical and biological properties, gold-based nanostructures that absorb in the NIR region, such as AuNR [122], AuNS [123], or AuNC [124], have frequently been applied in PTT. To optimize the effect of the nanoparticle-mediated PTT, selective localization within the tumor region at sufficient concentration is needed. This has been achieved using several approaches: (1) through the conventional Au-thiol surface bioconjugation to functionalize the surface with desired target molecules [54,121], (2) through a multifunctional nanoplatform that incorporated IONP allowing the IONP@AuNS to be directed into the solid tumors with an external magnet [53], and (3) through the use of macrophages as nanoparticle delivery vehicles [125]. Non-invasive imaging can also be used to guide and control PTT. Use of IONP@AuNS enabled MR thermal imaging (MRTI) to assess PTT by generating a real-time heat map during irradiation in order to control and optimize the applied thermal doses [126,127]. Initial result of a clinical trial using laser-excited AuNS (AuraShell) in combination with MR/ultrasound (US) fusion imaging to focally ablate low-intermediate-grade prostate have been shown to be safe and technologically feasible [123].

##### Hyperthermic Therapy

Hyperthermic therapy (HT) induces a mild rise in tumor temperature in the range of 40–50 °C, that can make cancer cells more susceptible to RT or chemotherapy [128,129,130]. Damage and death can also occur when cancer cells are exposed to HT for more than one hour [131]. Recent reports have shown the use of magnetic nanoparticles to induce hyperthermia to be very promising [132,133]. Magnetic nanoparticles allow the supply of tumor-specific hyperthermia intracellularly at the nanoscale level [134]. This can greatly improve the therapeutic efficacy of hyperthermia, concentrating the particles in the tumor and achieving better homogeneity [135]. An alternating magnetic field (AMF) is then applied, heating tumor cells to eradicate/damage cancerous cells.

## 3. Nanoparticles in Clinical Translation: Challenges Ahead

The many advantages brought about by the high functional versatility of inorganic nanoparticles in diagnostics and therapeutics motivates their continuous development and optimization as cancer nanomedicine. Nevertheless, despite the leaps achieved in preclinical research, very few have entered the clinic. Among all the inorganic nanoparticles, IONPs, with their biocompatibility and inherent magnetic properties that can be exploited in MR imaging, hyperthermic therapies and tumor ablation, have been the most explored clinically [136,137]. In fact, several of the IONP formulations have already been approved as MRI contrast agents for non-invasive diagnostic imaging. However, long term in vivo biocompatibility has shown some adverse effects, coupled with some suboptimal performance have resulted to market withdrawal (Table 1). Nevertheless, it is undeniable that inorganic nanoparticles have the potential to provide solutions to the gaps in current conventional cancer diagnostics and treatments. Recently, production of ferumoxtran-10 (Combidex) is being revived [138]. Ferumoxtran-10 works by differentiating benign and metastatic lymph nodes on a T2*-weighted IONP-enhanced MRI. Metastatic lymph nodes do not take up these nanoparticles while normal macrophages do, resulting in accumulation in normal lymph nodes after 24–36 h. Currently, no alternative contrast agent or medical device had comparable result with Combidex in prostate cancer [138]. This is one of the cases that highlight the unmet needs in clinical cancer diagnostics being bridged by inorganic nanomedicine.

On the case of nanoparticles being utilized as therapeutics, C dots have been the first optical inorganic nanoparticles approved by FDA as an IND for clinical trial in 2010 [86,139]. It is currently on Phase I/II for real time mapping of nodal metastases in several cancers (Table 2). AuNS are also being investigated in the clinic for NIR thermal ablation therapy (Auralase, AuraShell) [123,140] and AuNP as carrier of spherical nucleic acids to modulate Bc12L12 gene expression in recurrent glioblastoma (NU-0129) [141]. Nanotherm therapy, which consists of IONP with aminosilane shell that is introduced into the tumor and then heated under an alternating magnetic field, has recently obtained an investigational device exemption (IDE) approval from FDA to use as a focal ablation treatment in a clinical trial for intermediate risk prostate cancer [142]. Although there are several nanoparticles that have been in clinical trials, as of today, only Hensify^®^ (NBTXR3, Nanobiotix, Paris, France) is currently approved in the market (CE, Conformité Européenne).

The very low number of inorganic nanoparticles approved or under investigation for clinical use in comparison to preclinical research indicates significant challenges in moving from bench to the clinic. In August 2006, US FDA assembled a Nanotechnology Task Force to determine appropriate regulatory approaches and to identify and recommend mechanisms to address knowledge gaps in the adequacy and application of the current regulations. In July 2007, the task force concluded that nanotechnology combination products necessitate further exploration to ensure the regulatory pathways will yield predictable determinations in marketing the combination product whether as a drug, medical device, or biological product [143]. The increasing complexity of nanotechnology and its integration with other fields poses questions to the adequacy of existing regulatory frameworks, in particular with the assessment of the inherent risks of nanoparticles, including toxicity and human health impacts of exposures, effects of various exposure routes, and routes of administration. Hand in hand are the unintended effects of nanoparticles’ ability to cross physiological barriers (i.e., blood-brain barrier), and their long-term effects [144]. With the advancement of nanotechnology in human health, appropriate safety and efficacy requirements and risk-benefit measures that are tailored to characterize, assess, and report potential novel risks in nanotoxicity and exposure concerns are needed. In line with this, the National Nanotechnology Initiative and other federal agency collaborations are pushing for large-scale research efforts to characterize nanoscale materials and quantify their impact for purposes of developing toxicological assessment and testing tools [145]. In comparison with the traditional pharmaceutics, safety evaluation for each nanoparticle component is needed which results in more expensive trials [146]. FDA has established the Nanotechnology Characterization Laboratory (NCL) in collaboration with the National Institute of Standards and Technology (NIST) and the National Cancer Institute to perform preclinical efficacy and toxicity testing of nanoparticles. NCL can provide the infrastructure to accelerate the clinical transition of basic nanoscale particles at no cost to the investigators, lowering the barriers to researchers who aim to advance their research into the clinic [147,148].

## 4. Mechanisms of Nanoparticle Toxicity

It is important to establish the interactions of engineered nanoparticles with their biological effects in order to realize their full potential in the clinic. Understanding the mechanisms of their toxicities will lead to a more rational design that can overcome some of the major hurdles in their translation. In vitro assessment involves the current studies on the molecular and cellular mechanisms associated with nanotoxicity in relation to cellular binding and persistence, complement activation, oxidative stress, inflammation, and DNA damage while in vivo assessment deals with systemic toxicities of some nanoparticles, both known and unknown, on the human body. The systemic toxicity of gold- and iron-based nanoparticles, the two nanoparticle systems frequently used in cancer diagnostics and therapy, are highlighted in this section.

### 4.1. In Vitro Assessment

#### 4.1.1. Oxidative Stress, Inflammation, and DNA Damage

Nanoparticles induce reactive oxygen species (ROS) production due to the presence of pro-oxidant functional groups on their surface, which causes an imbalance in the redox state of the cell [152,153]. Excess ROS production causes oxidative stress and activates pro-inflammatory responses such as decreased mitochondrial membrane potential and decreased antioxidant enzymatic activity, among other effects [152]. These responses then lead to DNA damage and apoptosis as the end point of the nanoparticle-induced toxicity to cells. Smaller nanoparticles more easily penetrate through cell membranes, their higher surface areas and surface reactivities present greater cytotoxicity due to increased ROS production [153].

**Figure 2:**
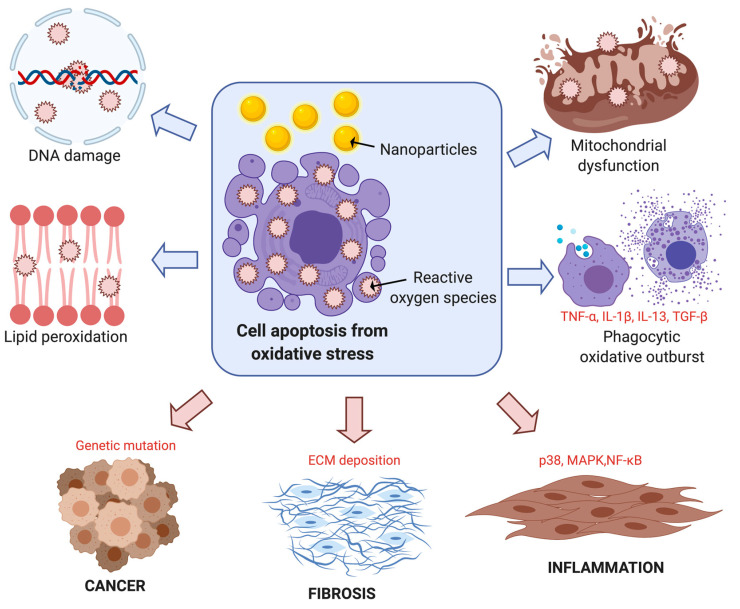
Prooxidant pathway for nanoparticle-induced toxicities. Upon nanoparticle exposure, ROS generation induces oxidative DNA damage, strand breaks, protein denaturation, and lipid peroxidation. Mitochondrial membrane damage results from excess free radical production, leading to necrosis and cell death. Phagocytes (i.e., neutrophils and macrophages) generate massive ROS upon incomplete phagocytosis of nanoparticle triggering an inflammatory cascade of chemokine and cytokine expression via activation of cell signaling pathways. Adapted from reference [154]. Created with BioRender.com.

In addition to increased production of ROS, nanoparticles induce cellular oxidative stress by depleting antioxidants that can combat mild oxidative stress [154]. Transcription and expression of antioxidant enzymes are regulated via nuclear factor Nrf2 induction. As the extent of oxidative stress increases, mitogen-activated protein kinase (MAPK) and NF-kB become activated as a pro-inflammatory responses [154]. However, excessive oxidative stress results in mitochondrial membrane damage and electron chain dysfunction, leading to DNA damage and eventually, apoptosis [154]. Silica or silicon dioxide nanoparticles (SiO_2_) were found to activate these oxidative stress-induced on human umbilical vein endothelial cells (HUVECs) [155]. The mRNA expression of Nrf2 and NF-kB was significantly upregulated [155], as illustrated in Figure 2.

Nanoparticles have been demonstrated to induce inflammatory responses in various cell types in different organ systems [152]. Some nanoparticles were recognized as pathogens by Toll-like receptors in the immune system, triggering increased production of inflammatory interleukins, chemokines, and adhesion molecules [155]. There is a strong link between inflammation and oxidative stress: inflammation potentially creates toxic by-products that promote the production of ROS while oxidative stress can result in the release proinflammation molecules, NF-kB and MAPK [152,154]. Research with SiO_2_ and TiO_2_ nanoparticles has indicated that inflammation through ROS generation can ultimately lead to changes in membrane permeability, leading to airway hypersensitivity reactions. Furthermore, the inflammatory and permeability effects have been proposed to extend beyond the lung and affect cardiovascular functioning as well [154]. Studies on specific molecular mechanisms associated with inflammation due to nanoparticle exposure are ongoing. Roy et al. found that inflammatory responses are linked to internalization of zinc oxide nanoparticles (ZnO) through endosome formation in macrophages, specifically by scavenger and caveolae pathways, in vitro [156]. The key inflammatory components studied included Cox-2 and iNOS expression, MAPKs, and cytokines such as IL-6, TNF-a, and IL-10. The results suggested that the inhibition of the caveolae internalization pathway reduces the expression of MAPKs [156].

DNA damage outcomes including, but not limited to DNA strand breaks, DNA protein cross-links, alkali-labile sites, and chromosomal aberrations, are observed upon oxidative stress induced by chronic exposure to nanoparticles [154]. Embryonic lung fibroblasts treated with AuNPs experienced DNA damage resulting to the formation of adducts with 8-hydroxydoxyguanosine and decreased expression of DNA repair and cell cycle checkpoint genes such as MAD2, cyclin B1, and cyclin B2 [152]. Regardless of the form of DNA damage, cells call upon repair mechanisms if the damage is reversible, or else transition into cell cycle arrest and apoptotic pathways. However, nanotoxicity has also been demonstrated to impact the DNA repair mechanisms themselves as seen in human embryonic lung fibroblasts exposed to AuNPs. In this case, DNA repair genes are downregulated and inability to repair the DNA damage would lead to apoptosis [152].

#### 4.1.2. Cellular Binding and Persistence

Cell-nanoparticle interaction studies allow for understanding of the cell adhesion, migration, and uptake pathways upon exposure to nanoparticles, both for drug-delivery efficacy and cytotoxic effects to healthy cells [157,158]. One key factor in the pathways of cell adhesion and migration is the doubling time of a particular cell line, as low doubling time indicates rapid proliferation and generally a higher migration efficiency [157]. The steps of cell migration include adhesion to the extracellular matrix, organization/disorganization of the actin cytoskeleton, membrane protrusion and retraction [157]. One particular study found that migration efficiency was dependent on the AuNP surface coating, but independent of size. The study proposed a potential mechanism in which cell adhesion to the nanoparticles occurs, then active migration and proliferation of cells and consequent cellular internalization sweeping out biocompatible nanoparticles in its way with non-biocompatible nanoparticles leading to cell toxicity [157].

Once cell adhesion and migration has occurred, the uptake component generally occurs through endocytosis, subdivided into phagocytosis and pinocytosis [158]. The key mechanisms of pinocytosis, which are involved in nanoparticle uptake include micropinocytosis, clathrin-mediated endocytosis, caveolae-mediated endocytosis, and clathrin- and caveolae-independent endocytosis [158]. Ultimately, most of their endocytic pathways lead to contents ending up in lysosomes for degradations [159]. However, in caveolae-mediated endocytosis, from endosomes, the contents form caveosomes, which are transported to the endoplasmic reticulum/Golgi apparatus, avoiding lysosomal degradation. This nuance in the uptake mechanisms was discovered as an important consideration for tailoring nanocarriers with drugs in cancer therapy [159].

Furthermore, the charge of the nanoparticles is critical in terms of cellular uptake and persistence [158]. Though previous studies have generalized the principle that positively charged nanoparticles interact more with cells compared to negatively charged ones due to the negative charge of the cell membrane, more current research indicates the greater complexity of this [158]. The protein corona is formed when nanoparticles are modified in a biological medium by various proteins that adsorb to the surface with different forces at play. Ultimately, studies have been mixed in terms of the effect of the protein corona on cellular uptake, with some indicating greater uptake with corona, while others demonstrated lesser uptake of nanoparticles. Thus, the rule of positive and negative charge on the nanoparticle surface cannot be simplified as such since there are multiple factors involving nanoparticle composition that affects cellular uptake [158].

#### 4.1.3. Complement Activation

The complement system and its activation are components of the body’s innate immune system against foreign invaders. Thus, upon exposure to nanoparticles, complement activation may induce inflammatory responses but in some cases, these can become uncontrolled posing a serious threat [160]. Complement activation may also be responsible for some allergic reactions caused by different nanoparticle-based therapies, including cancer therapy. There are three general complement pathways: classical, lectin, and alternative pathways which converge in the formation of SC5b-9 complex as the final activation product prior to the destruction of cells [160]. In addition, several complement proteins such as C1q, C3b, and C4b function as opsonins and specifically-tagged nanoparticles for rapid clearance. However, these various processes and components of the complement system are highly affected by the surface coating of the nanoparticles.

**Figure 3:**
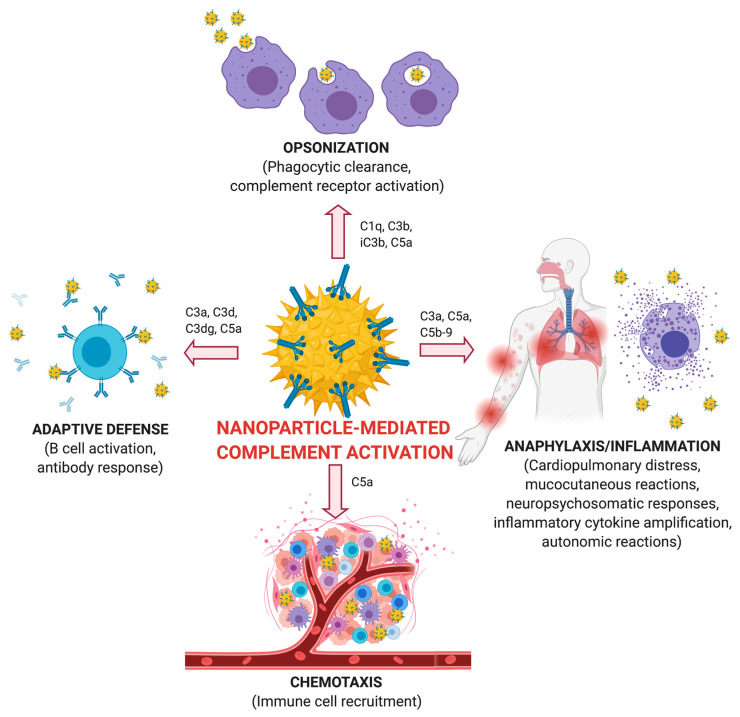
Key complement activation products in opsonization, anaphylaxis/inflammation, chemotaxis and adaptive defense resulting from nanoparticle-mediated complement activation. Created using BioRender.com.

Research on PEG-coated and citrate-capped AuNPs has indicated the differential effect on complement activation [161]. Ultimately citrate-capped AuNPs produced a size- dependent increase in the complement system end-product SC5b-9 in human serum, whereas the size-dependency was not present for PEG-coated AuNPs. Furthermore, PEG-coated AuNPs had a markedly reduced SC5b-9 level compared to citrate-capped, though it was still significantly increased compared with the control [161]. Another study with poly(2-methyl-2-oxazoline) (PMOXA) coated AuNPs demonstrated that this particular surface coating triggered complement activation to a greater extent only through the classical pathway [162]. The C1q mediated complement activation accelerated PMOXA opsonization and consequently, recognition by leukocytes and macrophages to a greater degree [162]. Greater clearance ability by the complement activation, without uncontrolled activation effects, would potentially decrease nanotoxicity as well. Complement activation effects are further summarized in Figure 3 as adapted from [160].

### 4.2. In Vivo Assessment

#### 4.2.1. Gastrointestinal

The gastrointestinal system is a complex, multi-organ system including the pharynx, esophagus, stomach, small and large intestines, rectum, liver, pancreas, and gallbladder. Nanoparticles have been employed in the treatment of various gastrointestinal cancers. The primary findings related to GI toxicity of nanoparticles depends on nanoparticle size and composition [163,164,165]. Specifically, studies on AuNPs have demonstrated that small particles (5 nm) preferentially produced pathological changes in the liver, whereas medium and large particles (20 nm and 50 nm) tended to target the spleen [163]. The toxic histopathological changes caused by the small AuNPs in the liver included steatosis, cytoplasmic degeneration, infiltration of inflammatory cells, Kupffer cells activation, and hemorrhage [163].

Further research on the cytotoxicity of AuNPs on different GI cancerous cell lines yielded variable results based on the composition of the conjugation or coating used. In a study by Huang et al. cell viability was greater than 90% in MGC803 gastric cancer cells after being exposed to AuNR@SiO_2_ targeted to folic acid, demonstrating that AuNPs by themselves were non-cytotoxic to MGC603 gastric cancer cells [164]. Similarly, another study by Li et al. incorporated chitosan AuNPs in esophageal cancer, which did not have any effect on benign human squamous esophageal epithelium cells or Barrett’s epithelium [165].

Another prominent type of nanoparticles used in biomedical applications are IONP or copper oxide (CuO) nanoparticles. The surface coating and size had a similar influence on overall toxicity patterns as demonstrated with AuNPs [166,167]. IONPs tend to accumulate in the liver and other RES organs. The iron products are recycled and then incorporated into pathways involved in hemoglobin, ferritin, and transferrin [166]. However, toxicity studies have indicated dose-dependent toxicity—high doses (2.5 mg/kg) could be potentially fatal as large aggregates formed quickly and rapid hemolysis can occur. When the anti-aggregating coating agent PEG was used with the IONPs, transient increases in the ALT enzyme was observed and there was much slower degradation and clearance of the PEG-coated IONPs [166]. Comparative toxicology research among SiO_2_, silver nanoparticles (AgNPs), and IONPs indicated the AgNPs caused a greater degree of GI systemic toxicity as demonstrated by increases in serum alkaline phosphatase and calcium, lymphocytic infiltration in the liver which was not observed in SiO_2_ and IONPs [167].

#### 4.2.2. Renal

Compared to the liver, the, kidneys tend to experience less nanoparticle, especially with AuNPs [168]. However, certain markers such as blood urea nitrogen, creatinine, protein, and globulin that have been found to be affected as a result of renal nanotoxicity. Studies have indicated a correlation between nanoparticle size and toxicity as 60 nm PEG-coated AuNPs demonstrated significant change in creatinine levels, indicating kidney toxicity, while sizes below 30 nm did not produce the same effect [169]. Within the kidneys, the proximal tubule epithelial cells were found to be the primary targets of nanotoxicity [170].

In one study, multiple cell lines from various organ systems, including the PK-15 cell line (epithelial porcine kidney) were studied for AuNP toxicity using dose-dependent and time-dependent measures [171]. At concentrations of 360 or 720 ng/mL, there was decreased cell growth within 24 h of addition. However, at concentrations below 360 ng/mL, the growth curves tended to shift back towards the control growth distribution and in some cases even exceed the baseline as time went on. This indicates that the toxic potential of gold nanoparticles at a reduced concentration are quite minimal and display some degree of reversibility as cell growth adapts and becomes resistant to change [171]. Histopathology of gold-related nanotoxicity indicated distorted glomeruli, mild necrosis, dilated tubules, and edema exudate. However, none of these findings were at the level of statistical significance as there was a great degree of variation among these minimal changes found in the renal system [171].

Comparison of renal toxicity effects of IONPs with AuNPs revealed similar trends: the distribution of iron in the kidneys was quite limited, with smaller particles being rapidly cleared in the urine [166]. At lower concentrations of IONPs, no significant change to the architecture of the kidneys occurs [172]. In addition, the coating further presented an effect on the renal toxicity potential. A study by Shukla et al. revealed that chitosan oligosaccharide-coated IONPs damaged kidney cell lines less, and showed less toxicity than bare IONPs in various cell lines, including Hek293 (human embryonic kidney), via MTT cell viability assay [173].

Overall, both AuNPs and IONPs appear to have limited cytotoxic impact on the renal system. However, modifications on the size, coating and concentration of the nanoparticles may enhance or decrease renal nanotoxicity as a result.

#### 4.2.3. Nervous

The nervous system is complex with many nuances that are not yet elucidated within the system itself, especially the brain. However, several research studies have examined aspects of nanotoxicity of AuNPs and IONPs, which will be the focus of this subsection.

Siddiqi et al. investigated changes in several biomarkers indicative of neurotoxicity upon injection of AuNPs in rat brains [174]. On one hand, there was a significance decrease in the enzyme glutathione peroxidase, which is an antioxidant in the brain that protects against oxidative damage. On the other hand, there were increases in markers of oxidative stress-derived DNA damage such as 8-hydroxydeoxyguanosine and heat shock protein 70 as well as apoptotic markers such as caspase-9. Furthermore, there was a significant increase in the neurotransmitters dopamine and serotonin, which demonstrated implications of AuNPs in potentiation of mood disturbances and chemical imbalances in the brain [174]. Other research has specifically studied the impact of AuNPs of different types on neural cells through the blood-retinal barrier [175]. In general, only small particles (less than 20 nm) were able to pass through the blood-retinal barrier and accumulate in the retinal layers. Furthermore, there was no toxicity demonstrated to neural cells such as retinal astrocytes, neurofilaments, and retinoblastoma cells in C57BL/6 mice [175].

The variability of toxicity effects on neural cells greatly depends upon the cell type and composition of the nanoparticles, which was demonstrated in other organ systems as well. For example, Joris et al. studied six different cell lines that included human and murine neural stem cells, human and mouse-derived progenitor cell lines, and human and murine neuroblastoma cell lines [176]. The study compared the relative toxicity effects of gold, iron oxide, and silver nanoparticles among these cell lines. Overall, it was found that AuNPs had the greatest degree of acute toxicity and IONPs having the lowest cytotoxic potential. In terms of the cell morphology, the C17.2 cell line was the only one with a reduction in cell area.

The cytotoxic potential of IONPs was further investigated on cultured neurons, astrocytes, and microglial cells. In rat cerebellar granular neurons, dimercaptosuccinate-coated IONPs accumulated within the cultured neurons by 1000-fold, but cellular integrity or viability was not adversely affected. This finding indicated the inherent potential of neurons to mediate oxidative stress effects through their antioxidant abilities [177]. While the finding with neurons was consistent with astrocytes, it did not hold true for microglial cells, which were rapidly damaged and displayed severe toxicity. At a mechanistic level, the rate of release of iron from internalized IONPs in microglial cells may be too rapidly transferred to lysosomes leading to toxic iron levels within the cells. Similarly, the cytotoxic potential of nanoparticles varies based on the particular mechanistic pathways associated with different nervous cell types to handle oxidative stress induced by the nanoparticles [176,177]. From a pathological perspective, accumulation of iron in the brain has been linked to neurodegenerative diseases such as aceruloplasminemia and neuroferritinopathy, as well as having the potential to play a role in Alzheimer’s and Parkinson’s diseases [178]. This research emphasizes the importance of finding the optimal amount of IONPs for therapeutic purposes in the nervous system.

#### 4.2.4. Cardiovascular

Cardiovascular nanomedicine has been employed in the diagnosis and treatment of cardiovascular diseases in addition to its role in cancer therapy [179]. One application of AuNPs in cancer therapy is the loading of these particles with doxorubicin (DOX, chemotherapy drug) for targeted drug delivery. The cardiotoxicity of these DOX-loaded and PEG-coated AuNPs have been studied in current pharmacological research [180]. Ultimately, DOX loaded onto AuNPs demonstrated no significant changes in cardiovascular function biomarkers such as serum lactate dehydrogenase (LDH) and creatinine kinase MB (CK-MB) levels compared to the free DOX. Similarly, another study further solidified these results in that DOX loaded on AuNPs did not create changes in CK-MB levels compared to baseline [181]. CK-MB is an enzyme in the myocardium that has served as the gold standard indicator of myocyte injury in many clinical and research settings [181].

There is limited research on the chronic cardiac toxicity of AuNPs, which was the aim of a study conducted by Yang et. al. on the effect of PEG-coated AuNPs at 2, 4, and 12-week time periods [182]. There was no significant decrease in the left ventricular ejection fraction across each time point for all sizes studied. Inflammatory mediators such as CD45+ and TNF-a indicated that chronic exposure to AuNPs did not spark inflammatory cell infiltration in the heart. Other studies that employed PEG-coated AuNPs have also concluded upon similar results that accumulation of AuNPs in the heart did not induce significant changes in cardiac hypertrophy, fibrosis or inflammation further demonstrating the strong biocompatibility of PEG-coated AuNPs in biomedical applications [183].

In the context of IONPs, cardiotoxic effects have been observed with IONPs in connection to myocardial damage due to iron accumulation. Unlike in the spleen, the macrophage clearance ability in the heart is limited, leading to longer-term accumulation not fully characterized in present research [184]. Particularly, some research has indicated that IV administration of IONPs resulted in a pro-coagulatory effect in vivo and in vitro while causing oxidative stress on the heart [185]. The effect of IONPs on different cardiac markers of oxidative stress in mice was also investigated. Data has indicated a significant increase of lipid peroxidation, reactive oxygen species, and superoxide dismutase in heart tissue compared with control groups [186].

Overall, there seems to be a certain degree of cardiotoxicity associated with IONPs, but the systemic effect of these microscopic changes is yet to fully be determined by current research. On the other hand, AuNP-related cardiotoxicity, in particular PEG-coated AuNPs, was quite limited. This demonstrates strong biocompatibility in terms of the cardiovascular system.

#### 4.2.5. Pulmonary/Respiratory

Nanoparticles are employed in different types of lung cancers as a method for targeted drug-delivery in therapy. Various research studies have examined the pulmonary toxicity associated with nanomedicine [187,188,189,190]. There seems to a degree of variability in the toxicity of AuNPs compared with other nanoparticle types, which appeared to be more consistent in their behavior [187,188]. A study by Avalos et al. compared the relative toxicity of silver- and gold-based nanoparticles on human pulmonary fibroblasts [187]. In general, the cytotoxic effect on the pulmonary fibroblasts was not size dependent for AuNPs, unlike some of the previous studies highlighted in sections on other organ systems. All sizes studied (30, 50, and 90 nm) demonstrated a reduction in cell mitochondrial activity and lactate dehydrogenase (LDH) leakage. Furthermore, in comparison to AgNPs, oxidative stress and production of ROS was greater with AuNPs in pulmonary fibroblast cells [188]. Another study examined three different human lung epithelial cell types (A549, BEAS-2b, and NHBE) for cytotoxic effects of AuNPs and AgNPs [188]. AuNPs were coated with either sodium citrate or chitosan, which created different surface charges on the particles. In general, A549 and BEAS-2B cells exhibited the least cytotoxic effects with an increase in LDH release only at the highest concentration of chitosan-coated AuNPs. However, NHBE cells were more affected in terms of cytotoxicity by AgNPs and AuNPs as measured by LDH release and membrane leakage [188].

Other researchers have investigated the cytotoxic effect of nanoparticles on key cells involved in the blood-air barrier in the pulmonary system [189,190]. In general, for different metal-organic frameworks, lung epithelial and alveolar macrophage cell lines were more adversely affected by lipid-coated nanoparticle systems [189]. Specifically, another study focused on an in vitro 3D lung model with three cell types of the epithelial tissue barrier: monolayer of alveolar cells, macrophages, and dendritic cells [190]. After initial exposure to AuNPs, there was no observable change in the cell morphology compared to control across the cell types. However, long-term effects are the current limitation of these research studies and remain unknown.

The general toxicity profile of IONPs in the pulmonary system is attributed to increased oxidative stress due to particle internalization, dissolution, release, and disruption of regular iron homeostasis [191]. In vivo studies have indicated that exposure to IONPs induces an elevated acute inflammatory response, which persists up to 28 days post-exposure. In addition, there was found to be an increase in the heat shock proteins and matrix metalloproteinases and evidence of progression to granulomas [191]. Additional research has focused on the toxic effects of metal nanoparticles following inhalation and intratracheal instillation as also are being used in the realm of targeted drug deliver in cancer [192,193]. After inhalational exposure of IONPs, there was a transient increase in acute total cell and neutrophil counts, pro-inflammatory chemokines, and oxidative stress in the initial time points. However, in the long-term there was no persistent inflammation at the end of the study demonstrating that IONPs had limited toxicity in the long-term [192].

Overall, while AuNPs appear to generally have adverse effects to the respiratory system, their significance in terms of long-term pulmonary cytotoxicity still varies depending on the type of AuNP and the particular lung cell type. Further studies are necessary to establish more generalized outlook on the effects of IONPs to various lung cells.

#### 4.2.6. Reproductive

Reproductive toxicity due to nanoparticles require further differentiation between their effects on male versus female reproductive systems. In general, nanoparticles have been demonstrated to cross the blood-testicle and blood-placenta barrier, pressing the importance of addressing reproductive toxicity [194]. Some studies have shown a decrease in sperm motility, albeit only at very high concentrations of AuNPs.

Female: Within the female reproductive system, nanoparticle accumulation tends to occur in the uteri and the ovaries [195]. In the case of AuNPs and IONPs, the greater accumulation within the uterus was observed for smaller nanoparticles. In addition, the negative impact on female sex hormones was largely seen in current studies with titanium nanoparticles (TiO_2_) as they increased expression of the Cyp17a1 gene which in turn increased estradiol, apoptotic-related genes, inflammatory and immune responses, among other effects [195]. Furthermore, some nanoparticles have shown to produce morphological changes in the follicles leading to a reduction in the mature oocytes present [196]. Ovarian toxicity was observed with long-term TiO_2_ nanoparticle, which caused a shapeless follicular antrum and irregular arrangement of cells, though, the results were inconclusive and not very general [196].

Male: In the male reproductive system, nanoparticle exposure tends to not only have an impact on the reproductive organs themselves, but also some potential effects on spermatogenesis and motility [195]. One particular study with AuNS found limited toxicity to the testes with no necrosis or histological disorganization within the germinal cells, spermatozoids, intertubular spaces, Leydig cells, and Sertoli cells in male mice [197]. On the other hand, additional research has indicated that exposure to zinc oxide nanoparticles (ZnO) presented with a reduction in testicular tissue and loss of cells in seminiferous tubules at an intraperitoneal dose of 250, 500, and 700 mg/kg/day [185]. Testicular toxicity due to ZnO has been established by several studies and some work has characterized the particular changes that manifest in the blood-testis barrier as well [196]. Mechanistically, ZnO have been suggested to trigger ROS, potentiate DNA lesions in germ cells, and downregulate the expression of gap junction proteins in the cell membrane [196]. Other researches have employed ligand-free and oligonucleotide-conjugated AuNPs to study toxic effects on spermatozoa specifically [198]. The findings indicated that sperm morphology and viability was generally not affected at any concentration [198].

In summary, within the male and female reproductive system, there is a limitation in current research on the cytotoxic potential of nanoparticles. The current studies seem to indicate that there are certain established adverse effects as those described in the spermatozoa and hormonal changes. However, the morphological changes to the reproductive organs and their long-term implications are highly dependent on the nanoparticle type, coating, and the cell type affected as with other organ systems [196].

#### 4.2.7. Immune

In the immune system, nanoparticle immunotoxicity refers to the adverse effects such as complement activation-mediated pseudoallergy, hypersensitivity, immunosuppression, and inflammasome effects [199]. In general, most nanoparticles tend to accumulate in organs of the mononuclear phagocytic system such as the liver and spleen. The immune cells that in turn produce toxic effects upon nanoparticle exposure include monocytes, platelets, leukocytes, dendritic cells, and macrophages [199].

Coating AuNPs with polyethylene glycol (PEG) avoided immunotoxic responses [199,200]. One study compared the immunotoxic effects of coating AuNPs with PEG and chicken ovalbumin (OVA) [200]. No significant cytotoxicity to RAW264.7 macrophages was observed at AuNP concentrations of 20 µg/mL. However, the uptake capacity for the OVA-AuNPs was greater compared to PEG-coated AuNPs. Additionally, PEG-coated AuNPs did not induce a significant increase in TNF-a, IL-6 and IL-1B for AuNPs larger than 35 nm. In general, small nanoparticles, despite differences in surface coating, appeared to present greater immunotoxic effects compared to larger ones [200]. AuNP toxicity in murine and human lymphocytes has showed overall viability was only significantly reduced at 200 µg/mL, but not any concentration below this [201]. Another key immune cell, dendritic cells have been targeted as points of entry for immunotherapeutic agents using AuNPs. Research has indicated that dendritic cells have very low cytotoxicity upon exposure to different sizes and concentrations of AuNPs [202]. However, small AuNPs with size of 10 nm displayed weak apoptotic effects in dendritic cells compared to larger nanoparticles. In terms of surface coating, positive charged-polymer-coated AuNPs did have a significant cytotoxic effect [202]. This indicates the necessity for correct identification of surface chemistry when designing biocompatible nanoparticles for cancer therapy and diagnosis usage, among other medical uses.

On the other hand, the immunotoxicity profile of IONPs is different. Within macrophages, RAW264.7 macrophages treated with IONPs demonstrated an increase in oxidative stress and an increase in cell proliferation within 24 h [203]. Further, another study indicated that murine and human macrophage cell lines exposed to PEI-coated IONPs induced the activation of toll-like receptor 4 signaling and ROS production via different pathways that in turn further increased the overall activation of macrophages leading to pro-inflammatory effects [204]. With B and T lymphocytes, the effect of IONPs remains unclear as initial studies indicated no effect on function and cell viability. While the lack of significant cytotoxicity still holds true with current studies, changes have been observed in T-cell function including delays in proliferation rate [205]. However, it has been demonstrated that compared to control at 13 weeks post-IONP injection, the distribution of B cells decreased while T cells increased. In addition, the number of dendritic cells increased, though surface markers for antigen presentation such as CD40 were decreased [206]. This suppression of antigen presentation in dendritic cells was a common feature indicated in other studies [150]. Thus, with dendritic cells, cytotoxicity was not a significant feature, but functional impairment was present.

Overall, the effects of nanoparticle toxicity on the immune system are complex and multi-faceted as it involves a variety of different cell types across organ systems. In general, the current research suggests that AuNPs present limited toxicity to the cells of the immune system including macrophages, lymphocytes, and dendritic cells. However, the results with IONPs s are more mixed, with some functional impairment effects demonstrated, though the cytotoxic potential to immune cells remains low. Additionally, the surface coating and electrostatic charge of the nanoparticles play a key role in immunotoxicity profile and potential evasion of the immune system responses in targeted drug-delivery systems, an area for additional research.

## 5. Strategies to Safe-by-Design Approach

**Figure 4:**
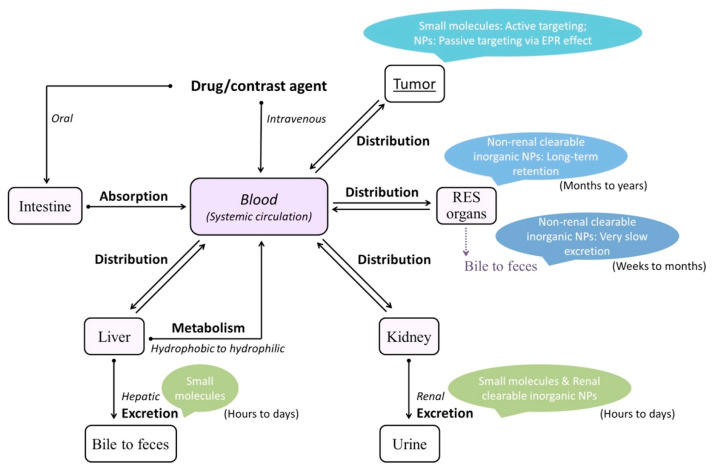
Pharmacokinetics includes absorption, distribution, metabolism, and excretion (ADME) of a drug or contrast agent. The four criteria influence the concentration of the substance and kinetics of the substance exposure to organs/tissues. For intravenous administration, the step of absorption is not involved because the substance is directly introduced to the systemic circulation. Reproduced from reference [211]. Copyright American Chemical Society, 2015.

As shown above, biological interactions of the nanoparticles and consequent effects both at the cellular and systemic levels are highly dependent on their physicochemical properties (i.e., size, shape, composition, surface charges and coating). To realize the optimal use of the nanoparticle platforms in the biological systems and to move forward with their clinical translation would require rational design that are driven by how these physicochemical properties could impact their fate and effects in the body (Figure 4). For example, it has been shown that inorganic nanoparticles with hydrodynamic size of sub-5 nm can be cleared out of the body efficiently through renal clearance and still maintain efficient tumor targeting in comparison with those of higher sizes which accumulate rapidly in the organs of the RES depleting their availability for the intended target and increasing the risk of toxicity with prolonged exposures [207,208,209,210,211]. At the same time, surface charge has been critical in non-specific binding and cellular uptake of the nanoparticles. AuNPs coated with amphiphilic polymers of varying surface charges resulted in positively charged nanoparticles having the highest uptake, however, this also led to higher toxicity [212]. Adsorption of serum proteins can be minimized by modifying the surface with zwitterionic or neutral organic coatings while also yielding small hydrodynamic size and high stability in biological media [208,213]. Toxicity inherent to the core composition of inorganic nanoparticles especially those consist of heavy metal atoms, and the leaching of ions from the dissolution of the nanoparticle core can be overcome by engineering the surface with a biocompatible coating. This strategy also regulates the high surface energy of the nanoparticles while providing stability, bioavailability and targeting [214]. With these findings, interest in the development of ultrasmall sub-5 nm nanoparticles, with judicious choices of surface coatings to improve biocompatibility and pharmacokinetics of nanoparticles has been rapidly rising [10,215,216,217].

However, nanoparticles are highly heterogeneous, with very diverse combinations of chemical composition, core-shell structure, shape, and functionalization. This poses a challenge in the experimental assessment of the relationship between their physicochemical properties and their toxicological effects. To overcome this, increasing reliance in in silico methods to improve the mechanistic understanding of nanotoxicity and develop computational models to predict outcomes to nanoparticle exposures and identify, assess, and classify their potential risks to human health in a cost- and time-efficient manner [218,219]. In silico nanotoxicology combines information technology with chemistry and biology in order to predict the toxicity of nanomaterials which provides an alternative testing method for the systemic investigation of large number of nanoparticles without animal testing [220,221]. This approach can be highly effective in gaining insight into the parameters that influence toxicity to predict the potential adverse effects of nanoparticles and thus impart proactive risk analysis and informed safe design [218].

### 5.1. Implementation of a Nanotoxicological Model

The most utilized tool in predictive nanotoxicology is the Quantitative Structure-Activity Relationship (QSAR), Quantitative Structure-Property Relationship (QSPR) [218]. In the context of nanomaterials, this is termed nano-QSAR [222] or Quantitative Nanostructure-Activity Relationship (QNAR) [223]. These predictive models aim to lessen the burden of testing by predicting risks based on the physicochemical properties of the nanoparticles, their intended application, and the available data [224]. No definite guidelines in dataset formation and model implementation in order to create a predictive model with nanotoxicological data have been set [219,221]. However, the Organisation for Economic Co-operation and Development (OECD) principle of a QSAR and in silico models in general requires a well-defined endpoint, unambiguous algorithm, defined domain of applicability, appropriate measure of goodness-of-fit, robustness and predictivity, and mechanistic interpretation [218]. The endpoint is defined as “a measure of activity for chemicals made under specific conditions” and refers to “any physicochemical property, biological effect or environmental parameter related to chemical structure that can be measured and modelled” [225].

**Figure 5:**
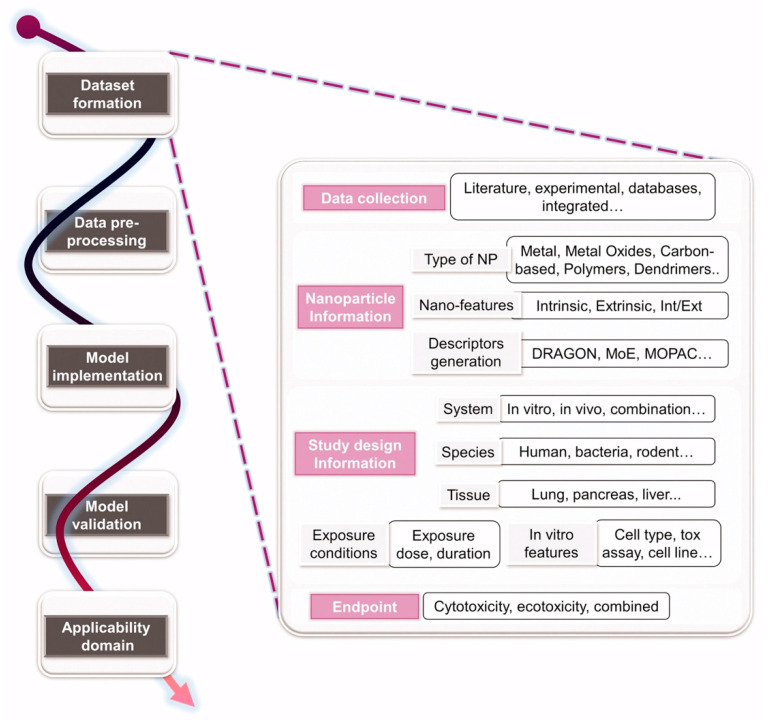
A summarized general roadmap for implementing a model in the field of nanotoxicology. The roadmap can be divided into five main parts: dataset formation review, data pre-processing, model implementation, model validation, applicability domain. Reproduced from reference [219]. Copyright Taylor and Francis, 2020.

Furxhi et al. laid out a general methodology for implementing a nanotoxicological model (Figure 5) by dividing it into five domains: (1) data set formation, (2) data pre-processing, (3) model implementation, (4) validation, and (5) applicability [219]. In order to form the data set, information on nanoparticles collected from existing literature, databases, and new experimental data, are recorded as inputs including nanoparticle type (composition: i.e., metal, metal oxide, carbon-based, etc.), and nanodescriptors, which are the experimentally determined physicochemical properties and theoretically calculated descriptors (i.e., quantum-mechanical descriptors, liquid drop model-based descriptors [226], full-particle descriptors [227]). Study designs such as testing system (in vitro, in vivo), species, organ tissues, and experimental conditions (i.e., dose and exposure) are also extracted. Finally, the toxicological endpoints which are used as the output to be predicted by the models are recorded. The most commonly used toxicological endpoints are in vitro assays since they are important indicators for biological evaluation. In vitro assays offer cheap, rapid, and reproducible tests that cover different cellular functions. Cellular viability is the most common predicted end point, followed by uptake. To predict combination of different endpoints, referred to as aggregated outcomes, weighting averages of the combined outcomes or ranking of hazard endpoints into a singular outcome is performed.

To optimize the performance of the model, the extracted data are then reduced by removing irrelevant or redundant information. Feature selection can be used by applying statistical performance metrics like genetic algorithm [222,228,229,230,231] and Pearson correlation coefficients [232,233,234,235] to select the optimal descriptors, thus avoiding overfitting training data and allowing the expert assessment of the mechanistic basis for the model [236,237].

Data normalization (z-score, min-max, log_10_) has also been used for variables with different scaling to reduce skewness of the data [238,239]. The model is then implemented using algorithms (i.e., trees, neural network, regression, rules, bayes, or meta algorithms) ensuring the full model structure and accurate model parameters are specified. The goodness-of-fit to measure how well the model accounts for variability, the robustness to measure the stability of model predictions when there is perturbation, and the predictability to assess model reliability are then tested.

### 5.2. In Silico Design and Study of Nanoparticles

The earliest nano-QSAR model was developed to evaluate the effect of chemical composition of 17 metal oxide nanoparticles on the cytotoxicity of Escherichia coli in terms of EC50 [222]. In this work, only one descriptor was used, which is the enthalpy of the gaseous cation formation, calculated through a quantum chemical PM6 method [240]. A strong correlation between the heat of formation and the cytotoxicity was established. The result was also in good agreement with experimental results identifying ZnO as one of the most toxic metal oxides, while TiO_2_ has one of the lowest toxicity [241,242]. Later on, recalculation of the nanodescriptor was performed based on SMILES (simplified molecular input line entry system), an intermediate between classical and nanodescriptors, generating the descriptor from molecular structure data, but can utilize experimental data related to the material and experimental conditions [243]. This proposed method is advantageous for its simplicity, doing away with building molecular models (i.e., only dependent on surface coating) [244,245]. However, not all compounds have SMILES notations, and the mechanistic interpretation of the model on the basis of the SMILES descriptors is limited [245].

Recently, a full-particle descriptor distinguishing the surface atoms (1 nm surface layer) from the core has been developed based on the force-field calculation of the potential energies of the whole nanoparticle [227]. This molecular model can calculate a set of 35 nanodescriptors on the features of the surface atoms relating to size, chemical composition, potential energy, topology, and lattice energy. In addition, due to the simplicity of calculation this full-particle descriptors can be effectively applied to QSAR/QSPR modeling for diverse nanoparticles as it does not need extreme computational resources.

An example of nanoparticle design utilizing this full-particle descriptors is the optimization of Fe-Doped ZnO to generate the best formulation that would release toxic metal ions to selectively kill cancer cells [246]. Here, they synthesized and characterized the particle size and crystallinity of a series of Fe-doped ZnO (0%, 1%, 2%, 4%, 6%, 8%, and 10% Fe), and determined their cytotoxicity in four cell lines, 2 of which are normal (murine mesenchymal stem cells and human bronchial epithelial cells, Beas-2B), and the other 2 are cancer cell lines (murine lung squamous carcinoma cells, KLN 205 and human cervical cancer cells, HeLa). The results of these experiments were correlated using one-parameter correlations with the full-particle nanodescriptors (i.e., size, average potential energy, no of Fe/Zn on the surface, surface area, lattice energy, etc.) generated from core-surface model of the spherical nanoparticles [227] calculated in the LAMMPS (large-scale atomic/molecular massively parallel simulator) software [247]. These descriptors were grouped according to their predictive power for the death rate of normal and cancer cells. Plot of the normalized representative descriptor values of the toxic subgroup (i.e., log of total number of Zn atoms in surface region of nanoparticle) and the non-toxic subgroup (i.e., log of total number of Fe atoms in surface region of nanoparticle) vs percent (%) Fe content resulted in the crossing between 2% and 3%. This indicates where the optimum % Fe value with the highest potency to kill cancer cells but with minimal damage to normal cells lies. In vitro validation in co-cultured normal and cancer cell lines, and in vivo therapeutic efficacy in DBA/2 mice subcutaneously injected with KLN 205 cells were in agreement with the predicted result from the nanodescriptor analysis.

Currently, improvement on the full-particle descriptors has been achieved. Based on the force vectors of all atoms and the polarizable model of oxygen atoms, calculation of the optimal thickness of the active surface layer of the nanoparticles has been fine-tuned and a new set of nanodescriptors relating to the highly active surface was obtained [244]. The new and improved nanodescriptors showed good correlation when used to model the cytotoxicity endpoint (i.e., cell death, membrane damage, ROS) of Fe-doped ZnO in HeLa and KLN 205 cancer cell lines. This overall approach can be applicable to any metal oxide nanoparticles with various dopings.

As the integration of computational modeling in the design of new nanomaterials progresses, there is a clear need for new universal nanodescriptors that can be used to characterize their diversity without the need for intense computational power. The applicability of the Delaunay tessellation approach, which was used in decomposing protein structures and protein–ligand bindings [248,249], to represent the nanostructures (i.e., to simulate the nanomaterial’s surface chemistry) and the use of Pauling electronegativity as empirical information to define descriptor characters have been explored [250]. The Delaunay tessellation approach decomposes the nanostructure surface into tetrahedra, in which vertices are atoms. The four atoms within a tetrahedron are uniquely selected such that their circumscribing sphere does not contain any of the other atoms [250,251]. Testing 191 unique AuNP with diverse biological activities and physichochemical properties in developing QNAR models validated the suitability of the obtained novel geometrical descriptors for quantitative modeling. Good predictabilities for both physicochemical properties (logP and zeta potentials) and nano-bioactivities (AuNP-enzyme bindings, cellular uptakes and ROS inductions) were achieved, confirming the utility of this modeling strategy as a universal tool to guide the rational design of nanomaterials [250].

In a subsequent study, cellular uptake and cytotoxicity endpoints were evaluated for three types of nanoparticles (Au, Pd, Pt), with two different sizes (i.e., 6 and 26 nm), and have six types of surface ligands of varying hydrophobicity [251]. Four experimental descriptors were determined (size, surface ligand density, zeta potential, and logP) and 126 nanodescriptors were calculated based on the Delaunay tessellation approach [250] and atomic electronegativity values. QNAR models were then developed by the *k*-nearest-neighbors approach (kNN), and validated by the leave-one-out (LOO) procedure (R^2^_kNN = 0.54). Ranking the top 10 nanodescriptors for the cellular uptake resulted in the experimental logP as the highest, indicating that uptake is highly modulated by the hydrophobicity of the nanoparticle core. AuNPs were determined to be more cytotoxic generating the highest level of oxidative stress in comparison with Pd of identical size, shape, and ligand. Pt, being the most hydrophilic, has less cellular uptake, and thus less cytotoxicity. These results confirm the importance of the nanoparticle core material when considering the design of nanomedicines.

## 6. Outlook: Data Driven Approach in Nanoparticle Design

**Figure 6:**
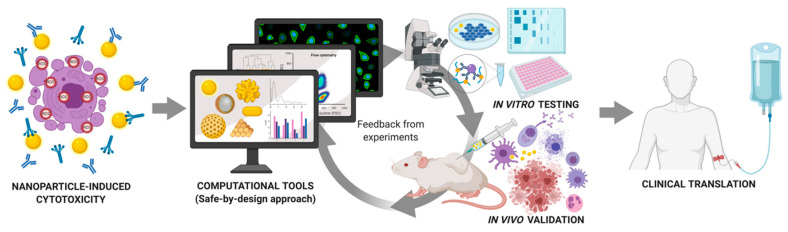
Data driven safe-by-design approach to tailor nanoparticle review for nanomedicine. Created with BioRender.com.

Therapeutic resistance leading to poor prognosis and clinical outcome of cancer patients have been often linked to intratumor heterogeneity [252]. As a result, finding the appropriate therapeutic regimen through conventional therapies can be challenging. In line with this, cancer nanomedicine can offer vast opportunities to tailor individualized treatments to patients. Inorganic nanoparticles with their multiple functionalities can help bridge unmet needs in early diagnosis and treatment to achieve potential breakthroughs in the clinic. However, the physicochemical variability of the nanoparticles and the flexibility in their design to tune their functionalities can result in complex biological interactions. This feature though highly advantageous for personalized treatments has become a hurdle to their clinical translation. Assessment of their biological interactions and effects on their safety profile can be challenging, inefficient and costly. In vitro and in vivo assays to evaluate safety profile and efficacy tend to be only valid in a case per case basis. As such, there is a need to implement a systematic approach to enable high throughput testing to enable risk assessment and clinical impact for a large number of nanoparticles to facilitate their practical use. Computational nanotoxicology and algorithm-based approaches to predict safety and efficacy of these nanoparticles are emerging. With the ongoing development and validation of computational tools, accurate in silico predictions with regards to safety and biological fate of the nanoparticle design can be achieved. Integration of computational modeling in the design stage (Figure 6) can be highly useful in their advancement and success in the clinic.

## Figures and Tables

**Table 1:** Inorganic nanoparticles approved in the clinic.

Name	Material/Functionality	Approved Application/Indication	Cancer Type	Approval Status
NBTXR3 Hensify^®^ (Nanobiotix, Paris, France)	50 nm crystalline hafnium oxide (HfO_2_) with phosphate coating	First-in-class radioenhancer	Locally advanced soft tissue sarcoma	CE Mark (2019)
Feridex I.V. (AMAG) Endorem	120–180 nm IONP colloid with low molecular weight dextran coating [149]	MR Imaging	Liver lesions	FDA (1996) Withdrawn (2008) Reasons: Hypotension, lumbar pain/leg pain, local pain, hypersensitivity [149,150]
Resovist	carboxydextrane-coated IONP, with a 45–60 nm hydrodynamic diameter [149]	MRI	Liver lesions	EMA (2001) Discontinued (2009) [151] Reasons: Vasodilatation and paraesthesia [149,150]
Ferumoxtran-10 /Combidex Sinerem (AMAG)	20–50 nm dextran coated IONP [138]	Imaging lymph node metastases	Prostate cancer	Only available in Holland, Discontinued Application withdrawn from EMA (2007) Application withdrawn from FDA (2005) [149,151]

**Table 2:** Inorganic nanoparticles undergoing clinical trials.

Name	Material/Functionality	Application/Indication	Cancer Type	ClinicalTrials.gov Identifier	Status	Concurrent Therapies/Interventions
NBTXR3 PEP503 (Nanobiotix, Paris, France)	50 nm crystalline hafnium oxide (HfO_2_) with phosphate coating	First-in-class radioenhancer	Pancreatic Ductal Adenocarcinoma	NCT04484909 Phase I	Recruiting (Est: December 2026)	
			Non-small cell lung cancer	NCT04505267 Phase I	Not yet recruiting (Est: March 2024)	
			Hepatocellular carcinoma	NCT02721056 Phase I/II	Unknown (Est: December 2018)	
			Advanced metastatic tumors	NCT03589339 Phase I	Recruiting (Est: March 2023)	anti-PD1
			Prostate adenocarcinoma	NCT02805894 Phase I/II	Recruiting (Est: November 2022)	Brachytherapy boost
			Locally Advanced Squamous Cell Carcinoma of the Oropharynx	NCT01946867 Phase I	Unknown (Est: June 2017)	
			Soft tissue sarcoma of extremity or trunk wall	NCT01433068 Phase I	Completed 2015	
			Soft tissue sarcoma of extremity or trunk wall	NCT02379845 Phase II/III	Active, not recruiting (Est: April 2020)	
			Head and neck SCC	NCT02901483 Phase Ib/II	Recruiting (Est: December 2020)	Cisplatin
			Rectal cancer	NCT02465593 Phase Ib/II	Recruiting (Est: June 2021)	5-FU, capecitabine, surgical resection (after neoadjuvant therapy)
AuraLase Therapy, AuroShell (Nanospectra Biosciences)	PEG-coated-AuNS	MRI/US fusion + Near-infrared thermal ablation therapy	Prostate cancer	NCT04240639 Extension study	Recruiting (Est: June 2023)	MRI/US guided laser irradiation
			Prostate cancer	NCT02680535	Active, not recruiting (Est: July 2020)	MRI/US guided laser irradiation
			Primary and/or metastatic lung cancer	NCT01679470 Pilot study	Terminated (Jun 2014)—limited study participants	Laser irradiation by bronchoscopic optical fiber
			Refractory or recurrent head and neck cancers	NCT00848042 Pilot study	Completed (August 2014)	
NU-0129 (Northwestern)	Spherical nucleic acid (SNA) on AuNP		Recurrent glioblastoma multiforme, gliosarcoma	NCT03020017 Early Phase I	Active, not recruiting (Est: September 2020)	
Magnablate	IONP	Magnetic thermoablation	Prostate cancer	NCT02033447 Early Phase I	Completed (January 2015)	Prostatectomy
NanoTherm (MagForce)	15 nm colloidal IONP	Magnetic thermotherapy	Glioblastoma Intermediate prostate cancer	Investigational Device Exemption		
Cornell Dots	Fluorescent cRGDY-PEG-Cy5.5-C dots	Real time mapping of nodal metastases	H&N, Breast, Colorectal cancers	NCT02106598 Phase I/II	Recruiting (Est: April 2021)	Fluorescence imaging, surgical resection
	64Cu-NOTA-PSMA-PEG-Cy5.5-C’ dot		Prostate cancer	NCT04167969 Phase I	Recruiting (Est: November 2021)	PET/MR imaging, surgical resection
	89Zr-DFO-cRGDY-PEG-Cy5-C’ dots		Malignant brain tumors	NCT03465618 Phase I	Recruiting (Est: March 2021)	PET imaging, surgical resection
Ferrotran^®®^ (Ferumoxtran-10)	Dextran coated IONP	Enhanced MRI	Prostate cancer	NCT04261777 Phase III	Recruiting (Est: December 2020)	MR imaging, surgical resection
			Pancreatic adenocarcinoma	NCT04311047	Recruiting (Est: December 2021)	MR imaging, surgical resection
Ferrumoxytol (AMAG, Inc., Waltham, MA, USA)	IONP	MR imaging contrast	Esophageal cancer	NCT02689401(PhI) NCT02857218(PhI) NCT02253602	Withdrawn 2016 Recruiting (Est. 2021) Completed 2018	Surgery, neoadjuvant therapy
			Prostate cancer	NCT01296139(PhI) NCT02141490(PhII) NCT00087347 NCT03358563(PhI)	Completed 2015 Completed 2018 Completed 2006 Suspended (Est. 2021)	Docetaxel, Degarelix, Bicalutamide
			Colorectal cancer	NCT01983371 (PhI) NCT03280277 (PhI)	Withdrawn 2016 Recruiting (Est. 2021)	
			Lung cancer	NCT03325166(PhII)	Recruiting (Est: 2022)	Pembrolizumab
			Brain neoplasms	NCT00978562 NCT03179449 (PhI) NCT03234309(PhII) NCT00659126(PhII) NCT00103038 NCT00769093 (PhI) NCT00660543 (PhI) NCT02466828 (PhI) NCT02359097 NCT03347617(PhII) NCT03264300 NCT02452216 (PhI)	Unknown Recruiting (Est. 2022) Recruiting (Est. 2022) Recruiting (Est. 2020) Active, not recruiting Terminated 2014 Completed 2014 Completed 2018 Recruiting (Est. 2023) Recruiting (Est. 2022) Recruiting (Est. 2022) Completed 2017	Surgery Carboplatin, bevacizumab Temozolomide qBOLD MRI Pembrolizumab
			Head and neck cancer	NCT01895829 (PhI) NCT01927887 NCT02479178(PhII)	Active, not recruiting Completed 2016 Terminated 2020	Surgery BIND-014 (docetaxel NPs)
			Breast cancer	NCT01770353(PhI) NCT00087347	Completed 2018 Completed 2016	MM-398 (Irinotecan NPs)
			Bladder cancer	NCT04369560(PhI) NCT02141490(PhII)	Recruiting (Est: 2022) Completed 2018	
			Pediatric cancers	NCT01542879 (Ph I/II)	Recruiting (Est: 2021)	18-FDG PET/MRI
			Pancreatic cancer	NCT02070705 NCT00920023(PhIV)	Recruiting (Est. 2021) Completed 2013	
			Bone neoplasms	NCT01336803(PhII)	Completed 2018	
			Soft tissue sarcoma	NCT01663090(PhI)	Withdrawn 2016	
			Any cancer with lymph node involvement	NCT01815333	Completed 2019	
			Solid tumors	NCT02631733 (PhI)	Suspended (Est. 2021)	Liposomal irinotecan, veliparib
